# Long-Term Effects of Developmental Exposure to Oxycodone on Gut Microbiota and Relationship to Adult Behaviors and Metabolism

**DOI:** 10.1128/msystems.00336-22

**Published:** 2022-07-07

**Authors:** Zhen Lyu, Robert R. Schmidt, Rachel E. Martin, Madison T. Green, Jessica A. Kinkade, Jiude Mao, Nathan J. Bivens, Trupti Joshi, Cheryl S. Rosenfeld

**Affiliations:** a Department of Electrical Engineering and Computer Science, University of Missouri, Columbia, Missouri, USA; b Department of Christopher S Bond Life Sciences Center, University of Missouri, Columbia, Missouri, USA; c Department of Biomedical Sciences, University of Missouri, Columbia, Missouri, USA; d Department of Genomics Technology Core Facility, University of Missouri, Columbia, Missouri, USA; e Department of Health Management and Informatics, University of Missouri, Columbia, Missouri, USA; f Department of MU Institute of Data Science and Informatics, University of Missouri, Columbia, Missouri, USA; g Department of Genetics Area Program, University of Missouri, Columbia, Missouri, USA; h Department of Thompson Center for Autism and Neurobehavioral Disorders, University of Missouri, Columbia, Missouri, USA; Drexel University

**Keywords:** opioids, behavior, brain, microbiota, gut dysbiosis, bacterial changes, gut microbiome, integrative correlation analyses, metabolism, rodents

## Abstract

Opioid drugs are commonly prescribed analgesic to pregnant women. Direct exposure to such drugs may slow gut motility, alter gut permeability, and affect the gut microbiome. While such drugs affect gut microbiome in infants, no study to date has determined whether developmental exposure to such drugs results in longstanding effects on gut microbiota and correspondingly on host responses. We hypothesized developmental exposure to oxycodone (OXY) leads to enduring effects on gut microbiota and such changes are associated with adult neurobehavioral and metabolic changes. Female mice were treated daily with 5 mg OXY/kg or saline solution (control [CTL]) for 2 weeks prior to breeding and then throughout gestation. Male and female offspring pups were weaned, tested with a battery of behavioral and metabolic tests, and fecal boli were collected adulthood (120 days of age). In females, relative abundance of *Butyricimonas* spp., Bacteroidetes, *Anaeroplasma* spp., TM7, *Enterococcus* spp., and Clostridia were greater in OXY versus CTL individuals. In males, relative abundance of Coriobacteriaceae, *Roseburia* spp., *Sutterella* spp., and Clostridia were elevated in OXY exposed individuals. Bacterial changes were also associated with predictive metabolite pathway alterations that also varied according to sex. In males and females, affected gut microbiota correlated with metabolic but not behavioral alterations. The findings suggest that developmental exposure to OXY leads to lasting effects on adult gut microbiota that might affect host metabolism, possibly through specific bacterial metabolites or other bacterial-derived products. Further work is needed to characterize how developmental exposure to OXY affects host responses through the gut microbiome.

**IMPORTANCE** This is the first work to show in a rodent model that *in utero* exposure to an opioid drug can lead to longstanding effects on the gut microbiota when examined at adulthood. Further, such bacterial changes are associated with metabolic host responses. Given the similarities between rodent and human microbiomes, it raises cause for concern that similar effects may become evident in children born to mothers taking oxycodone and other opioid drugs.

## INTRODUCTION

Pregnant women are commonly prescribed opioid analgesics that are highly addictive. Prescription opioid pain relievers were abused in 2016 by approximately 4% of the United States population (1). Opioid abuse is a primary noncommunicable, public health disorder in the United States (1) with oxycodone (OxyContin [OXY]) being one of the most and abused drug in this class. Opioid use disorder (OUD) is a particular problem in women of child-bearing age. OUD during pregnancy affects approximately 5.6 per 1,000 live births (2). More than 85% of pregnancies with women with OUD are left untreated (3). Infants prenatally exposed to opioids are at risk for neonatal abstinence syndrome (NAS or neonatal opioid withdrawal syndrome) (4). Maternal OUD is associated with poor fetal growth, potential premature birth, low birthweight, and possible congenital defects (5, 6). Infants with NAS have higher neonatal intensive care unit (NICU) admission rates and required longer hospitalization periods (7), resulting in greater health care costs for women with OUD and their infants (7). Even if infants with NAS appear healthy at birth, they may be susceptible to later diseases that a developmental origin of health and disease (DOHaD) origin (8, 9). Developmental exposure to opioid drugs might induce direct effects on offspring brain development and risk for later neurobehavioral disorders (10–17). An underexplored area though is that maternal exposure to opioids may alter the gut microbiome of her offspring and in turn lead to neurobehavioral changes due to affects through the microbiome-gut-brain axis. Brief gestation exposure, days 11 to 13 of gestation of mouse dams to the opioid, hydromorphone (10 mg/kg intraperitoneally) is sufficient to induce gut dysbiosis in her and her offspring (18). In mice, prenatal exposure to the opioid, methadone, induces analogous maternal and infant gut microbial changes (19). Scant amount is known about how early life exposure to OXY can lead to longstanding effects on gut microbiota and whether these are associated with adult behaviors and metabolism. We hypothesized that developmental exposure of mice to OXY induces long-term gut dysbiosis and such bacterial changes are linked to previously described neurobehavioral and metabolic alterations seen in adult offspring (20). To test this hypothesis, fecal boli were collected at adulthood from offspring exposed during the prenatal period to OXY or vehicle control (CTL), bacterial DNA isolated, and 16s rRNA sequencing was performed. Integrative correlation analyses were used to link bacterial alterations to our previously reported neurobehavioral results (20).

## RESULTS

### General microbiome features.

**FIG 1:**
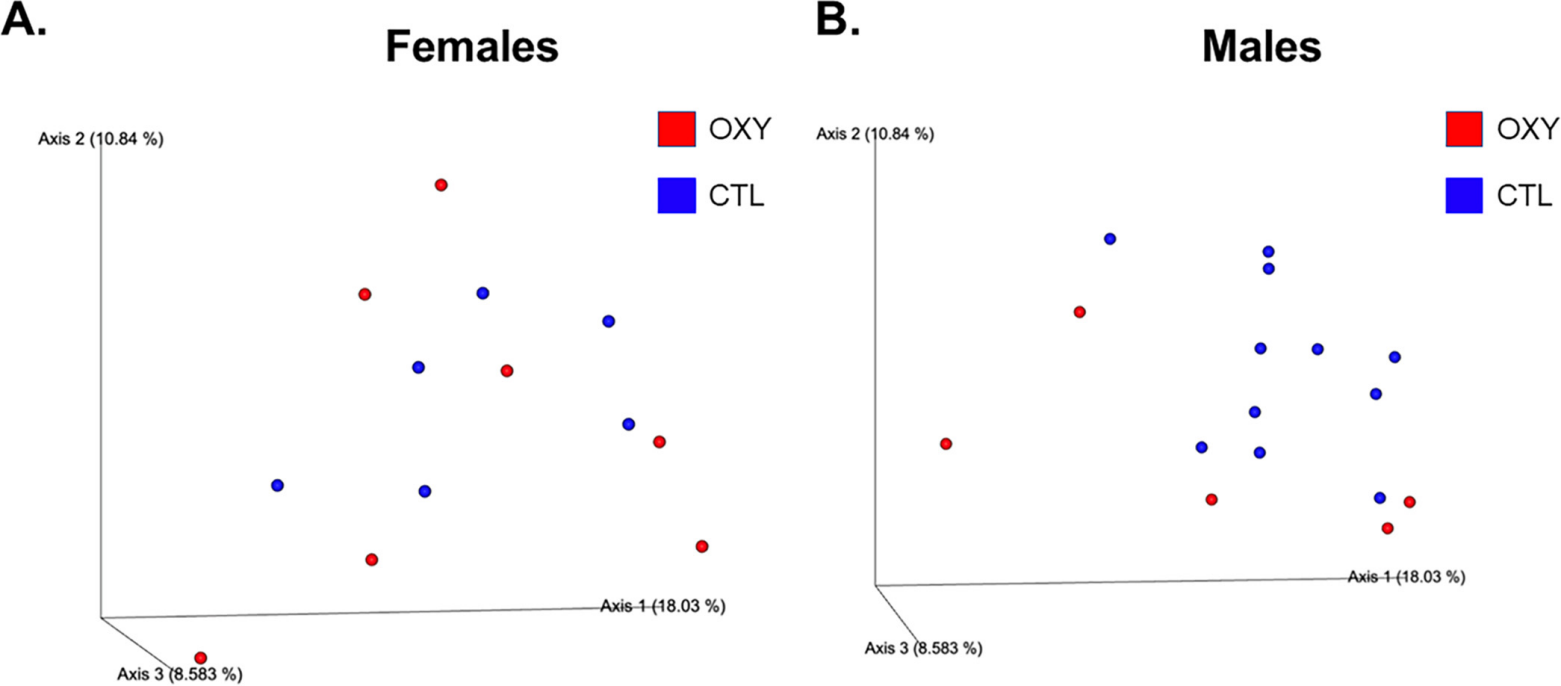
Three-dimensional PCoA plots to show β-diversity. PERMANOVA values for OXY exposed females versus CTL females and OXY exposed males versus CTL males 0.4 and 0.08, respectively. Replicates tested include *n* = 6 OXY exposed females, six CTL females, five OXY exposed males, and 11 CTL males.

We first considered whether OXY exposure affected overall α- and β- diversity. As determined by Simpson and Shannon, OXY exposure in females and males did not affect overall α-diversity relative to CTL (SAL) counterparts (Fig. S1). Based on the operational taxonomic unit (OTU) bar plot, no overt differences were observed in β- diversity based on offspring sex and OXY exposure (Fig. S2; Data Set 1). PCoA plot showed greater segregation between OXY and CTL male groups relative to PCoA plot comparing the results of these two groups in female progeny. PCoA plots revealed slight differences between OXY females versus CTL females and OXY males versus CTL males with PERMANOVA *P*-value for females and males 0.4 and 0.08, respectively (Fig. 1). The PCoA plots revealed no overall sex differences for CTL males versus CTL females and OXY exposed males versus OXY exposed females with PERMANOVA *P*-values of 0.1 and 0.7, respectively (Fig. S3).

10.1128/msystems.00336-22.1 FIG S1 *a*-diversity comparison of OXY exposed females and males and CTL females and males based on (A) Simpson diversity index and (B) Shannon. Download FIG S1, TIF file, 0.2 MB. Copyright © 2022 Lyu et al. 2022 Lyu et al. https://creativecommons.org/licenses/by/4.0/ This content is distributed under the terms of the Creative Commons Attribution 4.0 International license.

10.1128/msystems.00336-22.2 FIG S2 β-diversity as shown with OTU bar plot generated with QIIME 2. Key to the assorted colors representing individual OTU is included in File 1 (female and male data tabs). Replicates tested include *n* = 10 OXY exposed females, nine CTL females, eight OXY exposed males, and 12 CTL males. Download FIG S2, TIF file, 1.5 MB. Copyright © 2022 Lyu et al. 2022 Lyu et al. https://creativecommons.org/licenses/by/4.0/ This content is distributed under the terms of the Creative Commons Attribution 4.0 International license.

10.1128/msystems.00336-22.3 FIG S3 3D PCoA plots to show β-diversity. PERMANOVA values for CTL males versus CTL females and OXY exposed males versus OXY exposed females are 0.1 and 0.7, respectively. Replicates tested include *n* = 11 CTL males, six CTL females, five OXY exposed males, and six OXY exposed females. Download FIG S3, TIF file, 0.1 MB. Copyright © 2022 Lyu et al. 2022 Lyu et al. https://creativecommons.org/licenses/by/4.0/ This content is distributed under the terms of the Creative Commons Attribution 4.0 International license.

### Specific bacterial changes.

**FIG 2:**
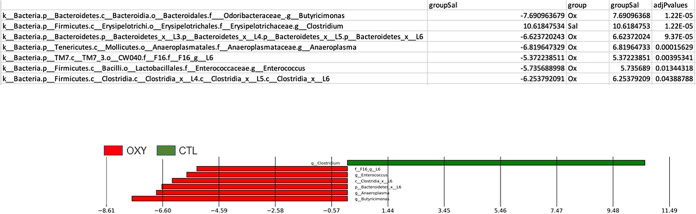
MetagenomeSeq analysis to determine bacterial differences between OXY females versus CTL females.

**FIG 3:**
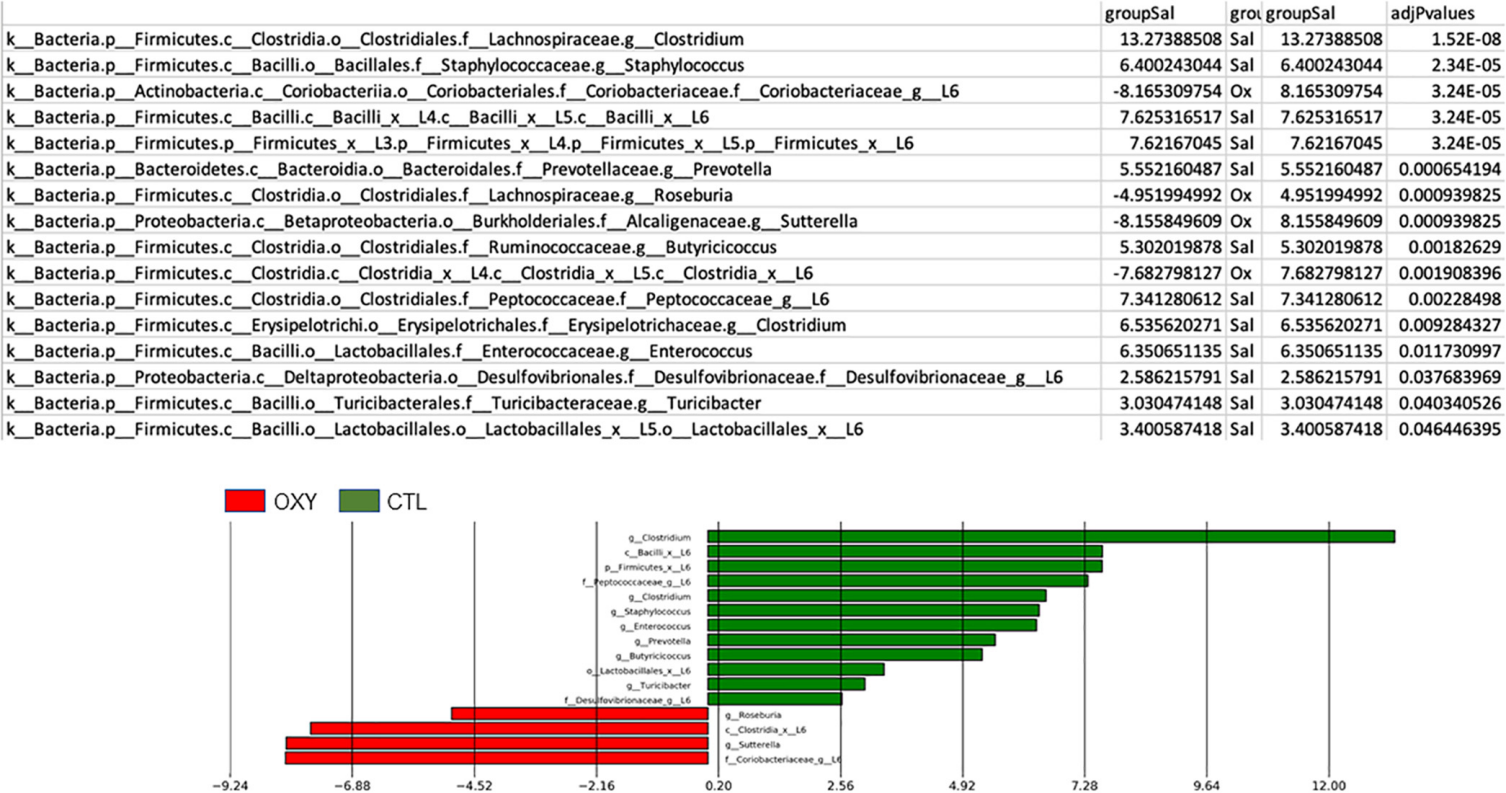
MetagenomeSeq analysis to determine bacterial differences between OXY males versus CTL males.

MetagenomeSeq was used to identify specific bacterial differences based on offspring sex and prenatal exposure to OXY or SAL control vehicle. In females, relative abundance of *Butyricimonas* spp., Bacteroidetes, *Anaeroplasma* spp., TM7, *Enterococcus* spp., and Clostridia were greater in OXY versus CTL (SAL) exposed individuals, whereas *Clostridium* spp. was greater in the CTL group (Fig. 2). Comparison of the male groups revealed greater number of bacterial differences. Relative abundance of Coriobacteriaceae, *Roseburia* spp., *Sutterella* spp., and Clostridia were elevated in OXY exposed individuals (Fig. 3). In contrast, *Clostridium* spp., Staphylococcus spp., Bacilli, Firmicutes, Prevotella, Butyricicoccus, Peptococcaceade, *Clostridium* spp., *Enterococcus* spp., Desulfovibionaceae, *Turicibacter* spp., and Lactobacillales had greater relative abundance in CTL versus OXY exposed individuals.

We also compared results in CTL males versus CTL females and OXY exposed males to OXY exposed females to determine if developmental exposure to OXY affected sexually dimorphic differences in gut microbiota. Relative abundance of *Corynebacterium* spp., *Anaeroplasma* spp., Firmicutes, *Clostridium* spp., Bacteroidetes, and Bacilli was greater in CTL males versus CTL females, whereas Coriobacteriaceae was increased in CTL females compared with CTL males (Fig. S4). In contrast, relative abundance of Corynebacterium and Coriobacteriaceae was greater in OXY exposed males versus OXY exposed females. Peptococcaeceae, *Enterococcus* spp., *Prevotella* spp., *Butyricicoccus* spp., Staphylococcus spp., and Staphylococcaceae (Fig. S5.)

10.1128/msystems.00336-22.4 FIG S4 MetagenomeSeq analysis to determine bacterial differences between CTL males versus CTL females. Download FIG S4, TIF file, 0.2 MB. Copyright © 2022 Lyu et al. 2022 Lyu et al. https://creativecommons.org/licenses/by/4.0/ This content is distributed under the terms of the Creative Commons Attribution 4.0 International license.

10.1128/msystems.00336-22.5 FIG S5 MetagenomeSeq analysis to determine bacterial differences between OXY males versus OXY females. Download FIG S5, TIF file, 0.3 MB. Copyright © 2022 Lyu et al. 2022 Lyu et al. https://creativecommons.org/licenses/by/4.0/ This content is distributed under the terms of the Creative Commons Attribution 4.0 International license.

10.1128/msystems.00336-22.6 DATA SET 1 List of all OTUs identified female and male groups. Female data are included in the first tab, and male data in the second tab. These tables are generated based on MetagenomeSeq analyses. Both tabs include *P* values, adjusted *P* values (false discovery rate, FDR), and count data. Those highlighted in green are significant at an FDR ≤ 0.05 and were then used to generate the correlation (circos) plots shown in Fig. 6 and 7 for females and males, respectively. Those highlighted in yellow were also significant at an FDR ≤ 0.05 but were not included in correlation plots as we were able to ascertain more information in terms of Linnean classification. In other words, those shown in green have additional Linnean classification details, such as genus and species, relative to those highlighted in yellow. Download DATA SET 1, XLSX file, 0.03 MB. Copyright © 2022 Lyu et al. 2022 Lyu et al. https://creativecommons.org/licenses/by/4.0/ This content is distributed under the terms of the Creative Commons Attribution 4.0 International license.

### PiCRUSt analysis.

**FIG 4:**
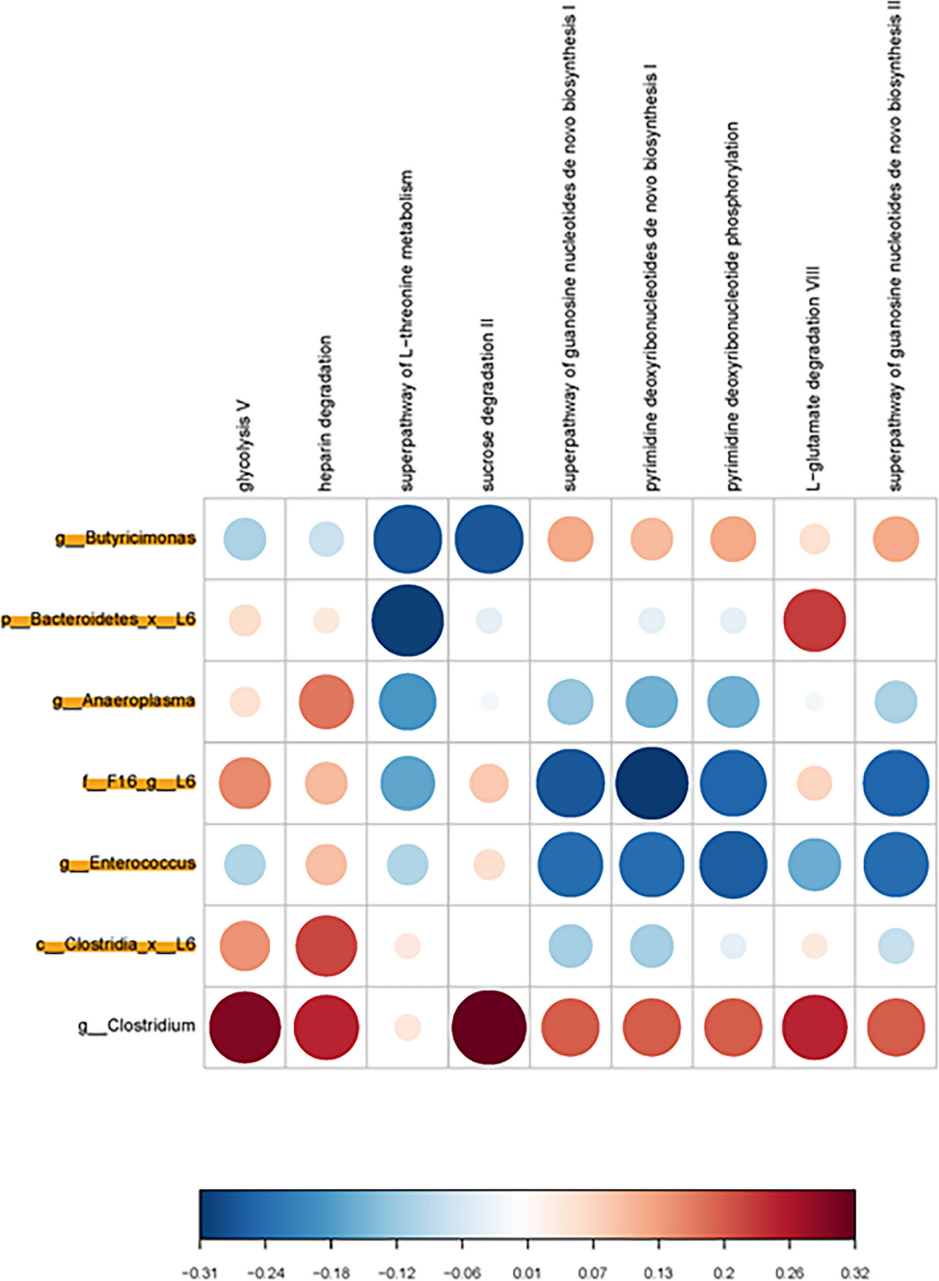
Bacterial metabolic and other pathway differences in the fecal microbiome of OXY exposed females versus CTL females. As described in Fig. 7 of Ma et al. (51), correlations between the PICRUSt-generated functional profile and QIIME II-generated genus level bacterial abundance were calculated and plotted against treatment group. Those genera that were identified by MetagenomeSeq as being different between the two groups are depicted. Bacteria that are highlighted had increased relative amounts in OXY exposed group. Metabolic pathway designations are delineated at the top of the figure. Shading intensity and size of the circles indicates the Kendall rank correlation coefficient between matrices. Red indicates a positive correlation, whereas blue designates a negative correlation. Red squares surrounding the circles are indicative of a *P* value ≤ 0.05, although none were present for this comparison. Legend for the quantitative scores associated with the range of blue to red colors is listed below the figure. Legend for the scores associated with the range of blue to red colors is listed below the figure.

**FIG 5:**
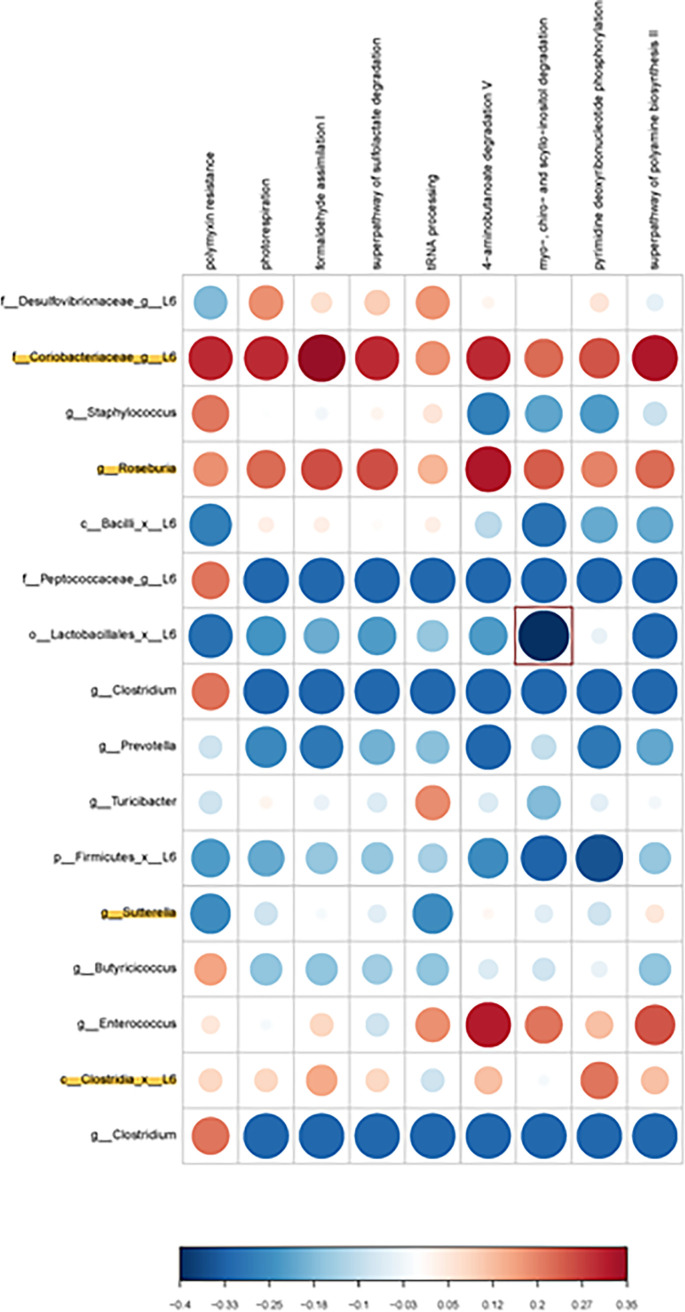
Bacterial metabolic and other pathway differences in the fecal microbiome of OXY exposed males versus CTL males. As described in Fig. 7 of Ma et al. (51), correlations between the PICRUSt-generated functional profile and QIIME II-generated genus level bacterial abundance were calculated and plotted against treatment group. Those genera that were identified by MetagenomeSeq as being different between the two groups are depicted. Bacteria that are highlighted had increased relative amounts in OXY exposed group. Metabolic pathway designations are delineated at the top of the figure. Shading intensity and size of the circles indicates the Kendall rank correlation coefficient between matrices. Red indicates a positive correlation, whereas blue designates a negative correlation. Red squares surrounding the circles are indicative of a *P* value ≤ 0.05, although none were present for this comparison. Legend for the quantitative scores associated with the range of blue to red colors is listed below the figure. Legend for the scores associated with the range of blue to red colors is listed below the figure.

PiCRUSt analysis was done to examine potential metabolic pathways that might be altered based on the identified bacterial changes. While no metabolic pathways reached statistical significance in the females, the ones that showed the strongest positive correlation where glycolysis and sucrose degradation with *Clostridium* spp. (Fig. 4). Conversely, those that showed the greatest negative correlation in females were l-threonine metabolism with Bacteroidetes and purine deoxyribonucleotides *de novo* biosynthesis with TM7. In males, the one inverse correlation that was statistically significant at *P* < 0.05 was myo-, chiro-, and scyllo-inositol degradation with Lactobacillales (Fig. 5). Positive correlations that approached significance were Coriobacteriaceae with polymyxin resistance, phospho-respiration, formaldehyde assimilation, superfamily of sulfoacetylation, 4-aminobutanoate degradation, and superfamily of polyamine biosynthesis. Firmicutes showed a strong trend to being inversely correlated with pyridine deoxyribonucleotide phosphorylation.

### Integrative correlation analyses.

In previous studies, we found that developmental exposure to OXY resulted in socio-communication deficits that persisted from weaning through adulthood (20). Such offspring also had cognitive impairments, reduced voluntary physical activity, and weighed more than CTL counterparts. In the hippocampus, OXY-exposed offspring had altered expression of genes encoding opioid receptors and those involved in serotonin signaling. We did not, however, detect any signs of substance abuse or dependency, although this was not explicitly tested in this prior work.

**FIG 6:**
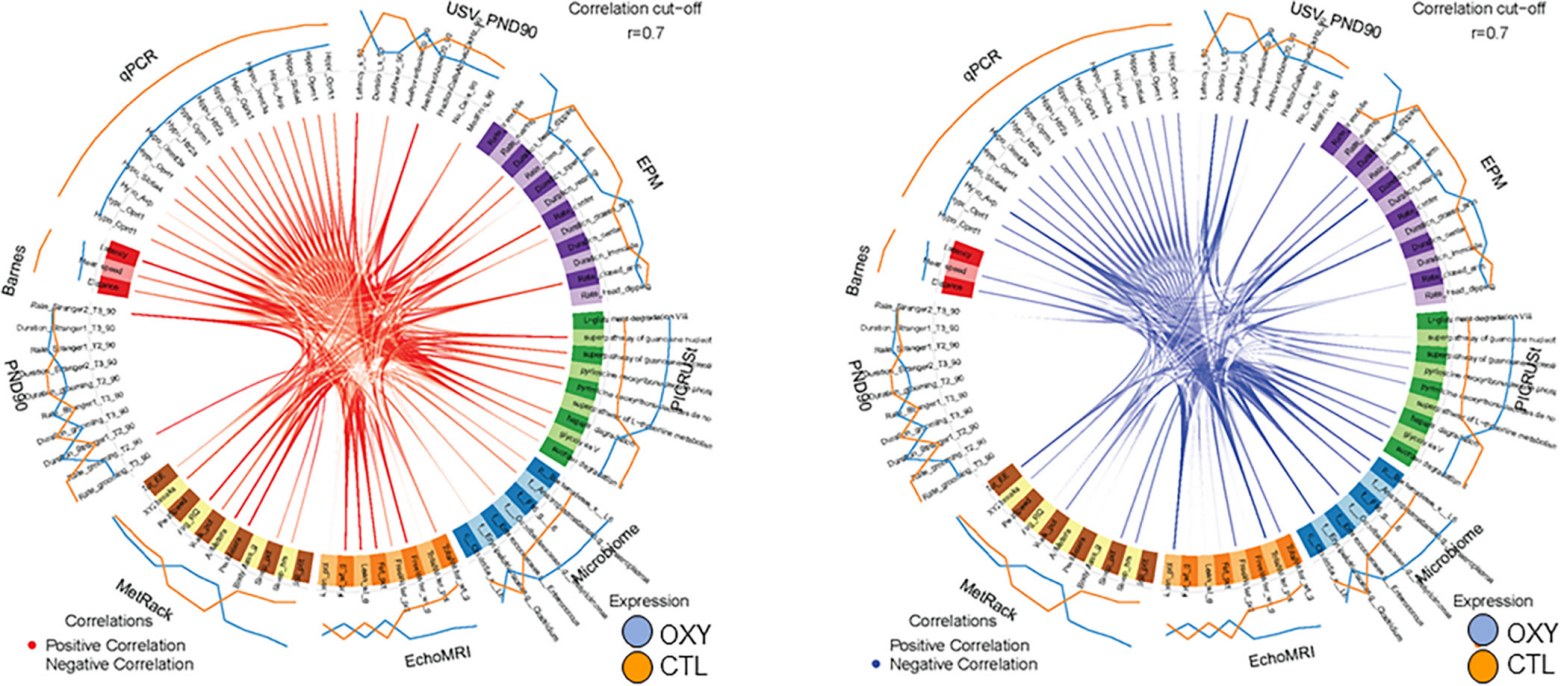
Circos plot correlations between gut bacterial, metabolites, and behavioral and metabolic parameters in OXY exposed females versus CTL females. Red lines in the center indicate a positive correlation. In contrast, blue lines indicate a negative correlation. Results for CTL (SAL) females are indicated with an orange line outside the circle. Blue line indicates results for OXY females. The color of the line further from the circle indicates the group where these results are greater.

**FIG 7:**
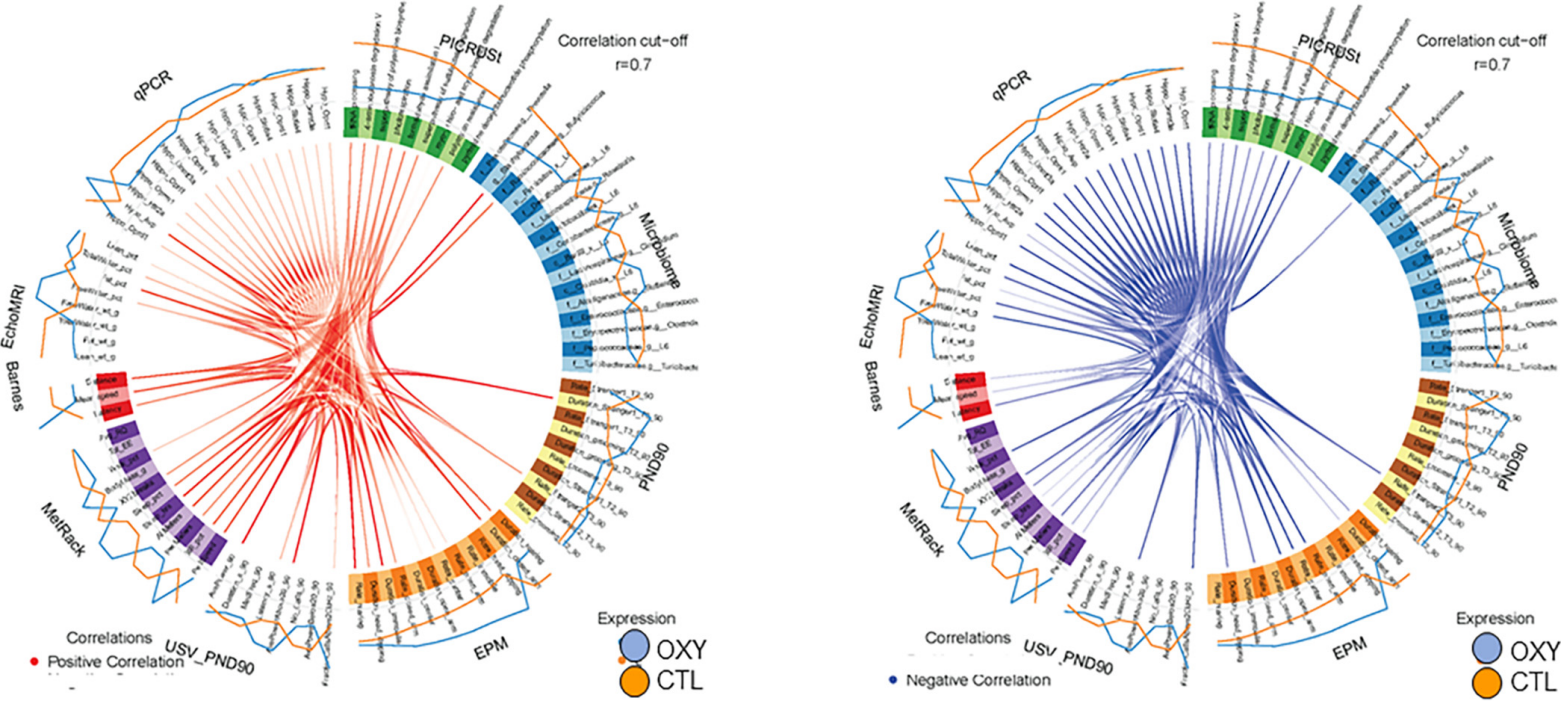
Circos plot correlations between gut bacterial, metabolites, and behavioral and metabolic parameters in OXY exposed males versus CTL males. Red lines in the center indicate a positive correlation. In contrast, blue lines indicate a negative correlation. Results for CTL (SAL) males are indicated with an orange line outside the circle. Blue line indicates results for OXY males. The color of the line further from the circle indicates the group where these results are greater.

As these were the same offspring used in this previous study (20), we next used mixOmics analyses with a ≥ 0.70 correlation value, which is considered stringent, to examine for associations between gut microbiota changes and these adult parameters. This approach revealed several positive and negative correlations relative to gut microbial changes in both female and male groups. In females, Bacteroidetes, *Anaeroplasma* spp., and *Butyricimonas* spp. positively correlated with fat weight (Fig. 6). *Clostridium* spp. was positively associated with PedMeters (total meters walked while in the indirect calorimetry unit). Conversely, Bacteroidetes, *Anaeroplasma* spp., and *Butyricimonas* spp. were negatively linked with lean percentage and total energy expenditure (EE) in the indirect calorimetry unit. *Clostridium* spp. was inversely associated with walking percentage in the indirect calorimetry unit and mean speed and distance traveled in the Barnes maze. In males, Prevotella and Staphylococcus spp. were positively associated with mean speed in the Barnes maze (Fig. 7). Butyricicoccus was inversely correlated with total water percentage. The one significant metabolic pathway in males, myo-, chiro-, and scyllo-inositol degradation, negatively correlated with total water and lean percentages.

## DISCUSSION

The goals of the current study were to investigate whether developmental exposure of mice to OXY results in persistent changes in gut microbiota that extend into adulthood and are sex-dependent. Second, we sought to determine whether such gut microbiota changes might be linked to changes in metabolic pathways and previously identified neurobehavioral and metabolic disruptions reported at adulthood in male and female offspring prenatally exposed to OXY (20). Past mice studies have shown that OXY exposure can lead to direct maternal and infant effects (18, 19). However, the current study is the first to explore whether such exposure can result in long-term gut dysbiosis changes. In females developmentally exposed to OXY, the primary gut bacterial changes were relative abundance of *Butyricimonas* spp., Bacteroidetes, *Anaeroplasma* spp., TM7, *Enterococcus* spp., and Clostridia. *Butyricimonas* spp. are Gram-negative and anaerobic bacteria associated with butyrate production (21, 22). Women with nonalcoholic fatty liver syndrome (NAFLD) have also been reported to have an increase in this bacterial genus (23). Rats treated with the Antidepressants fluoxetine or amitriptyline also show greater relative abundance of *Butyricimonas* spp. (24). Humans with Parkinson’s disease who show mild cognitive impairment (PD-MCI) have elevations in this bacterium (25). *Butyricimonas* spp. relative numbers are greater in a cohort of patients with autism spectrum disorder (ASD) relative to children without this disorder (26). Taken together, the collective findings suggest that other pharmaceutical agents may influence the abundance of this bacterium that has been linked to metabolic and neurobehavioral diseases.

Bacteroidetes relative abundance is also greater in simian immunodeficiency virus (SIV)-infected rhesus macaques treated with morphine (27). In mice, proliferation of Enterococcus faecalis correlates with gut dysbiosis due to morphine treatment (28). Nosocomial infections with Clostridium difficile (CDI) have been disproportionately seen in chronic opioid users (29). Another study found that the odds risk ratio was significantly elevated in those taking opioid drugs (30). Such findings are supported by a preceding study (31). The primary caveat to our current findings relative to previous reports is that our bioinformatics analysis only revealed that it was a Clostridia that was elevated in the OXY exposed groups, and thus, it could be a pathogenic or nonpathogenic microorganism. Further studies, including metagenome sequencing are needed to further characterize and type the specific bacteria identified based on this initial screening. The above studies are based on direct exposure to opioids, whereas the females used in this study were only exposed prior to birth. Notwithstanding, our results in light of past findings suggest that opioids might induce similar bacterial changes regardless of exposure window.

Gut bacterial changes in OXY exposed male offspring differed from those of their female siblings. However, Clostridia was elevated in male and female offspring developmentally exposed to OXY. Male offspring also had increased relative abundance of Coriobacteriaceae, *Roseburia* spp., and *Sutterella* spp. Relative reductions in *Clostridium* spp., Staphylococcus spp., Bacilli, Firmicutes, Prevotella, Butyricicoccus, Peptococcaceade, *Clostridium* spp., *Enterococcus* spp., Desulfovibionaceae, *Turicibacter* spp., and Lactobacillales were noted in these males. *Roseburia* relative abundance has been shown to be greater in the gut microbiota of pregnant women with ketonuria (32). Butyrate production by *Roseburia* may increase serum ketone levels in both the mother and her fetus. Maternal obesity during pregnancy may also increase this bacterium (33). Another pregnancy cohort study revealed that the abundance of Staphylococcus relative to Clostridium, *Roseburia*, and Coriobacteriaceae were positively correlated with fasting blood glucose (34).

Reduced relative abundance of *Roseburia* has been reported in humans on opioid drugs (35). Firmicutes is reduced but *Prevotella* relative abundance was found to be increased in the above study testing effects of opioids on rhesus macaques (27). In a study with African American men, interrelationships were found between type 2 diabetes mellitus, metformin treatment, and opioid usage for Bifidobacterium and *Prevotella* genera (36). Maternal exposure to the opioid, methadone, resulted in similar bacterial changes in the mother and her offspring with Lachnospiraceae NK4A136 genus being one of the primary genera associated with several of the shared features (19). This group though did not show changes in relative abundance in OXY exposed male or female offspring. Differences in the previous studies testing effects of opioids and the current results could be attributed to generational effects, type of opioid, and age of offspring examined (birth versus adulthood).

Based on the bacterial changes identified in female and male offspring, our prediction was that PICRUSt would reveal potential alterations in short-chained fatty acids, including butyric and propionic acid. However, such predictions were not identified. Instead, pathways enriched, although they did not reach statistical significance, in female offspring were carbohydrate and threonine metabolism and nucleotide biosynthesis. The one metabolic pathway that was significant in males and correlated with Lactobacillales, which showed relative reductions in OXY exposed males, was myo-, chiro-, and scyllo-inositol degradation. Inositols are polyol that exist in different stereoisomers, e.g., myo-inositol, d-chiro- and scyllo- forms. Supplementation of such compounds may hold therapeutic promise for a variety of diseases, especially neurological disorders. Provisioning of a mouse model for AD with scyllo-inositol reduced plague formation within the brain (37), decreased amyloid load, and astrocyte activation in the cortex (37, 38). In the current study, decrease in the overall abundance of Lactobacillales might suggest an increase in degradation of these inositols with resulting reductions in such stereoisomer forms. Based on the above studies, our prediction that such reductions would correlate with neurobehavioral outcomes. However, the mixOmics analyses only revealed an inverse correlation with metabolic phenotypes, lean and total water percentages. Follow-up metabolomic analyses using various mass spectrometry approaches are needed to confirm these predictions.

While no study to date has examined whether developmental exposure to opioids affects later metabolomic profiles, a few studies considered effects of direct exposure on the brain metabolome profile. Metabolome screening of the cerebrum in mice treated with tramadol revealed changes in metabolites associated with oxidative damage, inflammation, and disruption of the GABA neurotransmitter system (39). In rhesus macaques, chronic morphine exposure results in neural alterations in neurotransmitters and metabolites associated with membrane and energy metabolism (40).

While some of the neurobehavioral and metabolic changes in the offspring might be ascribed to alterations in the gut microbiota, they could also be due to direct effects on fetal brain development as OXY and other opioid drugs can readily cross the placenta (41, 42), be taken up by placental cells (trophoblasts) (43), and transferred to the fetus where it can affect fetal brain development. In previous work, we have shown that OXY exposure can affect mouse placental morphology and gene expression patterns (44) that in turn might also the initial stage of fetal brain growth through the placenta-brain axis (45).

The limitations of the current study are that we did not collect fecal samples from the dam to determine whether direct and developmental exposure to OXY induces similar changes. It would also have been of interest to examine and compare gut microbiota changes throughout the life span in exposed male and female offspring. However, our primary goal of these studies was to link those gut microbiota changes identified at adulthood with behavioral and metabolic alterations at this time. Future studies thus include examining gut microbiota throughout the life span in the presence and absence of opioids, such as OXY, as well as determining the microbial composition from the F0. We also seek to determine whether other opioid drugs, including agonistic and antagonistic agents, have similar effects as OXY. While 16s rRNA sequencing provides generation information on bacteria that might be affected by this opioid, it does not indicate how this drug affects bacterial genomes. For this reason, we will use metagenome seq in future studies. As detailed, future work will also examine the effects of developmental exposure to opioids on host and bacterial metabolites by using a variety of metabolomic approaches.

In conclusion, these studies are the first to show that developmental exposure to OXY alters the gut microbial profile at adulthood. The specific bacteria affected by this opioid show sex-dependent differences. Relative abundance of Clostridia was elevated in both males and females exposed to OXY. Bacterial changes were also associated with predictive metabolite pathway alterations that also varied according to sex. In both males and females, affected gut microbiota correlated with metabolic but not behavioral alterations. The findings suggest that OXY induced changes in the gut microbiota might affect host metabolism, which could be through specific bacterial metabolites or other bacterial-derived products. Further work is needed to characterize how developmental exposure to OXY affects host responses directly and through the gut microbiome.

## MATERIALS AND METHODS

### Animals and treatments.

Current animal experiments were approved by our Institutional Animal Care and Use Committee (ACUC, Protocol #9590). All studies conformed to the NIH Guidelines for the Care and Use of Laboratory Animals. Seven-week-old male and female CF1 mice were ordered from Envigo (Madison, WI), and females were habituated to the animal facility for 1 week prior to being placed on one of two treatments. Mice were maintained on a 12-h light: 12-h dark cycle. The average room temperature is 70°F, and the humidity range is between 30% and 70%. Female mice were randomly assigned to be in the OXY (Catalogue # O1378; Sigma Chemical, St. Louis, MO) or saline CTL groups. At 8 weeks of age, the OXY group received 5 mg OXY/kg body weight in 0.9% saline with an average volume of 0.1 mL injected intraperitoneally (IP) between in the morning daily for 2 weeks prior to breeding and then throughout gestation. During this time, the CTL group received comparable IP injection volumes of 0.9% saline. Females were weighed weekly throughout the course of the experiment, and the dose of OXY was adjusted to continue to provide a dose of 5 mg/kg. This dose and route of administration (IP) was used based on past findings that showed such concentrations mimic those achieved in humans with OUD (46–48). No ill effects were noted in mice treated with OXY or saline control IP injections. No differences in appetite or weight gain were noted for females in the OXY or saline control group. The treatments commenced 2 weeks prior to breeding to include the periconceptional period, as this may be important in preimplantation embryonic development (49, 50). Animals were provided food and water *ad libitum* and fed an AIN93G phytoestrogen-free diet (Envigo, Madison, WI) to reduce any exogenous estrogen exposure.

### Breedings.

After 2 weeks of being treated daily with OXY or CTL solutions, females were paired with potential CF1 breeder males and examined the next morning for a vaginal plug. The day a vaginal plug was observed was considered E 0.5. If no vaginal plug was observed in the morning, males were placed in separate cages and repaired that evening with females. Female mice were maintained on their respective treatments until parturition. No differences in fertilization rates or pregnancy success rate were noted between the two maternal groups. One male and one female offspring from each litter (*n* = 13 male and 13 females for CTL and 10 male and 10 female mice for OXY group) were randomly chosen to undergo behavioral and metabolic testing. The same male and female offspring from each litter were used for all of the behavioral and metabolic assessments and gene expression studies.

### Collection of fecal samples and isolation of fecal microbial DNA.

Same sex siblings were housed together until the time of fecal collection. At 120 days of age, each animal was placed in a cage alone without any bedding. Four to five fecal boli were collected from each animal and placed in sterile 2 mL cryogenic vials (Corning Incorporated, Corning, NY) and placed in liquid N_2_ until they were transferred to a −80°C freezer. Thereafter, the samples were stored until they were used for bacterial isolation and gut microbiota analysis. The fecal microbial DNA was isolated from a portion of the fecal boli collected using the Invitrogen Pure Link Microbiome DNA purification kit (Thermo Fisher Scientific, Waltham, MA) and in accordance with the manufacturer’s protocol. The quantity of DNA isolated was measured using Qubit 3.0 Fluorometer (Life Technologies, Grand Island, NY). The number of replicates tested is comparable to other studies examining how *in utero* environmental changes can affect gut bacterial populations and have shown that such sample sizes can result in statistical differences between offspring groups (51, 52).

### 16S rRNA sequencing.

The University of Missouri (MU) DNA Core Facility prepared bacterial 16S ribosomal DNA amplicons from extracted fecal DNA by amplification of the V4 hypervariable region of the 16S rDNA with universal primers (U515F/806R) flanked by Illumina standard adapter sequences (53, 54). The rest of the procedures were performed as described previously (55, 56). The resulting amplicon pool was analyzed by using the Advanced Analytical Fragment Analyzer automated electrophoresis system, quantified with a Qubit fluorometer using a quant-iT HS dsDNA reagent kit (Invitrogen), and diluted according to Illumina’s standard protocol for sequencing on the MiSeq.

Paired-end Illumina MiSeq DNA reads were joined, combined, and imported from Casava 1.8 paired-end demultiplexed fastq format to QIIME2 (57) format using the “qiime tools import” method from QIIME 2. Samples having less than 200 reads were excluded from the further analysis as the few reads typically represent transcriptional noise. This retains for 10 female samples for OXY group, nine female samples for saline control group, eight male samples for OXY group, and 12 male samples for saline control group. For the sequence quality control, the “Deblur” plugin in QIIME2 was utilized to filtering the sequences with –p-trim-length assigned 120. The rooted phylogenetic tree and unrooted tree were created using the “qiime phylogeny align-to-tree-mafft-fasttree” command.

### Bioinformatics analyses.

Microbial diversity was evaluated by running alpha diversity and beta diversity on the OTU tables. Diversity analysis was conducted for male and female groups using the q2-diversity plugin from QIIME2. The diversity comparisons were assessed between OXY and saline control for male and female group. For alpha diversity, Simpson diversity index (a quantitative measure of community richness) and Shannon diversity index rarefaction plots were generated using the “qiime diversity alpha-rarefaction” command supported by the QIIME2. Measurements of beta-diversity were facilitated by the QIIME2 command “qiime diversity core-metrics-phylogenetics” with p-sampling-depth assigned with 41,980. The permutational MANOVA (permanova) value was calculated using “qiime diversity beta-group-significance” command. For the taxonomic analysis, we used a pretrained Naïve Bayes classifier and the “q2-feature-classifier” plugin. This classifier was created based on the Greengenes 13_8 99% OTUs.

There were initially 10 OXY exposed females, nine CTL females, eight OXY exposed males, and 12 CTL males prior to filtering out samples with low reads quality, and criteria based on the PCoA analysis program. Therefore, for the PCoA analysis and subsequent analyses, there remained six OXY exposed females, six CTL females, five OXY exposed males, and 11 CTL males.

MetagenomeSeq (58) was used to determine the OTUs that are differentially abundant between OXY and saline CTL groups, CTL males versus CTL females, and OXY exposed males versus OXY exposed females. This program first creates the MRexperiment object taking the OTU table and metadata as input. The cumNormStatFast and cumNorm functions were used to calculate the normalization factors and normalized count matrices. After taking care of normalization, it utilized the fitZig (Zero-inflated Gaussian mixture model) function to detect the significant differentially abundant OTUs with *P*-value < 0.05.

### Functional metagenomics predictions.

Bacterial metabolic characterization of sample types was facilitated with q2-picrust2 (59–64) (the phylogenetic investigation of communities by reconstruction of unobserved states). It uses “qiime picrust2 full-pipeline” command to get Enzyme Commission (EC), Kyoto Encyclopedia of Genes and Genomes Orthologs (KEGG), and MetaCyc pathway prediction. The final output files, including EC, KEGG, and pathway prediction in QZA format. To export the result, “qiime tools export” command was used to convert the QZA file to BIOM format, then use “biom convert” to convert BIOM file to plain-text for downstream analysis.

DESeq2 (65) was used to highlight the pathway terms that are significantly differentially abundant between OXY and CTL saline group. DESeqDataSetFromMatrix function was first used to create the experiment object with count matrix and metadata file. The differential expression analysis was estimated by DESeq function based on the Negative Binomial (a.k.a. Gamma-Poisson) distribution. This function first calculates the size factors and dispersion and then apply the Negative Binomial GLM fitting and Wald statistics test. Shrunken log_2-fold_ changes (LFC) and SE was added to the results table from DESeq using lfcShrink function. Finally, filtering the significant terms with adjusted *P*-value < 0.05.

### Correlation of taxa abundance and metabolic activity abundance.

To correlate the taxa abundance with metabolic characteristics of sample types, a custom R script provided as a gift from Dr. Jun Ma and Kjersti Aagaard-Tillery, Baylor College of Medicine, Houston, TX was used (51), as we have done previously (55, 66, 67). In these figures, the correlation of the abundance of taxa (from the OTU table) with the predicted metabolic function (from MetaCyc pathways as determined by q2-picrust), was calculated with the R stats function cor.test (https://cran.r-project.org/), using the Kendall method, a rank-based measure of association. The cor. test function outputs the correlation coefficient and significance of a comparison of an OTU with a pathway term across samples. The matrix of all the correlation values was visualized using the R package corrplot (https://cran.r-project.org/). The area and intensity change together so that larger, darker, circles represent correlation coefficients that are larger in magnitude. The scale to the right of each figure relates those shades of color to the value of the correlation coefficient.

### Multiomics integrative correlation analyses.

The mixOmics (68) R package was used to correlate the bacterial genera changes simultaneously with body composition, brain histological data, and behavioral results, which enabled the integration of the microbiome, behavioral (social testing, ultrasonic vocalization, Barnes maze, and elevated plus maze, EPM), metabolic phenotyping (EchoMRI and MetRack) and qPCR gene expression results that have been previously reported (20). We conducted sparse discriminant analysis with partial least square regression with function “block.splsda.” The circos plot was generated by using the “circosPlot” function with correlations calculated using the method from González et al. and 0.7 correlation was used as the cutoff (69).

### Data availability.

All the raw sequencing data are available at BioProject link: https://www.ncbi.nlm.nih.gov/bioproject/PRJNA786078.
