# Supplementary figures and images for: Long-Term Effects of Developmental Exposure to Oxycodone on Gut Microbiota and Relationship to Adult Behaviors and Metabolism
Source: mSystems. 2022 Jul 7;7(4):e00336-22. doi: 10.1128/msystems.00336-22 (PMC9426609; doi:10.1128/msystems.00336-22)

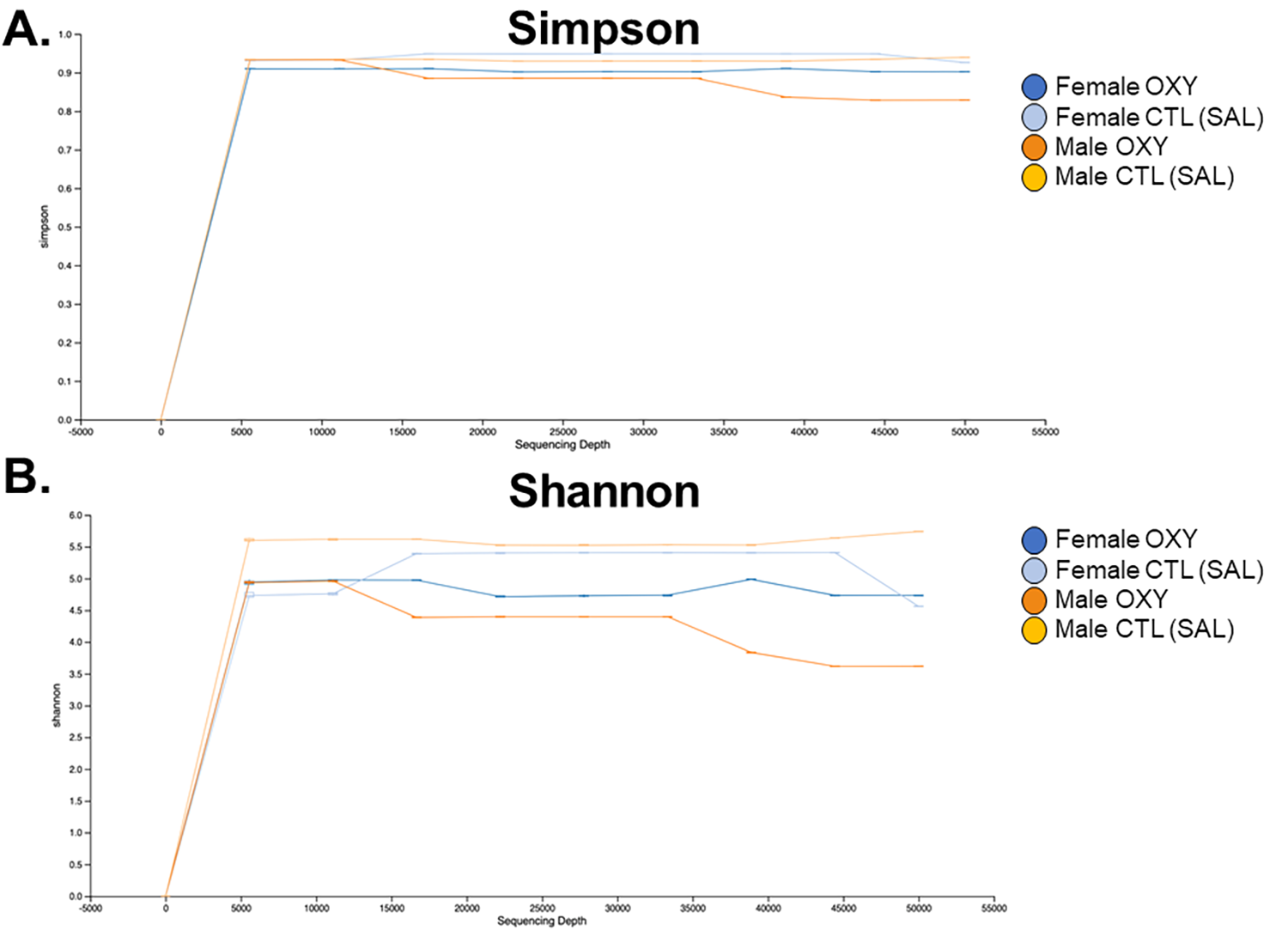

Supplement: FIG S1 [file msystems.00336-22-sf001.tif]

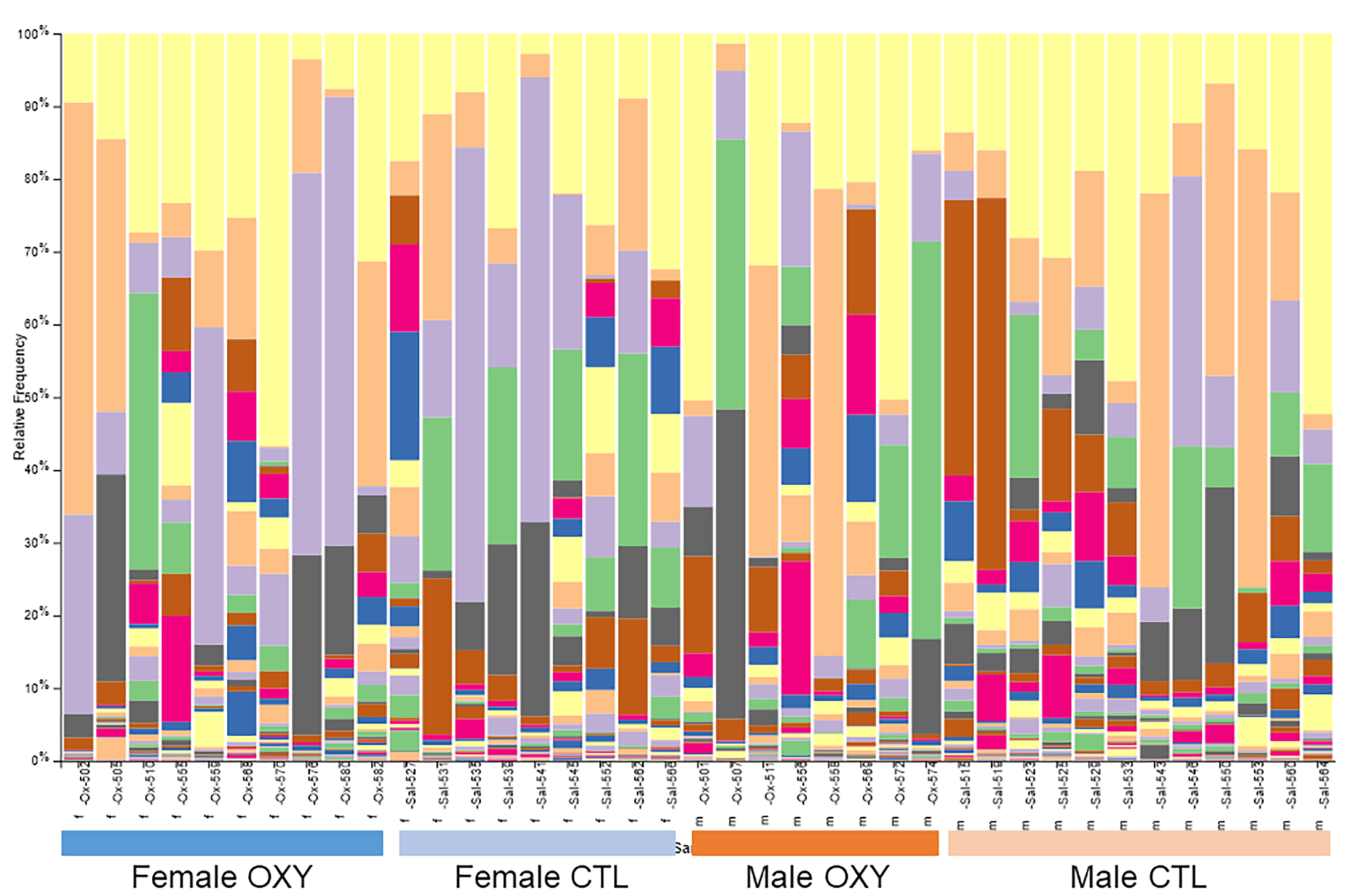

Supplement: FIG S2 [file msystems.00336-22-sf002.tif]

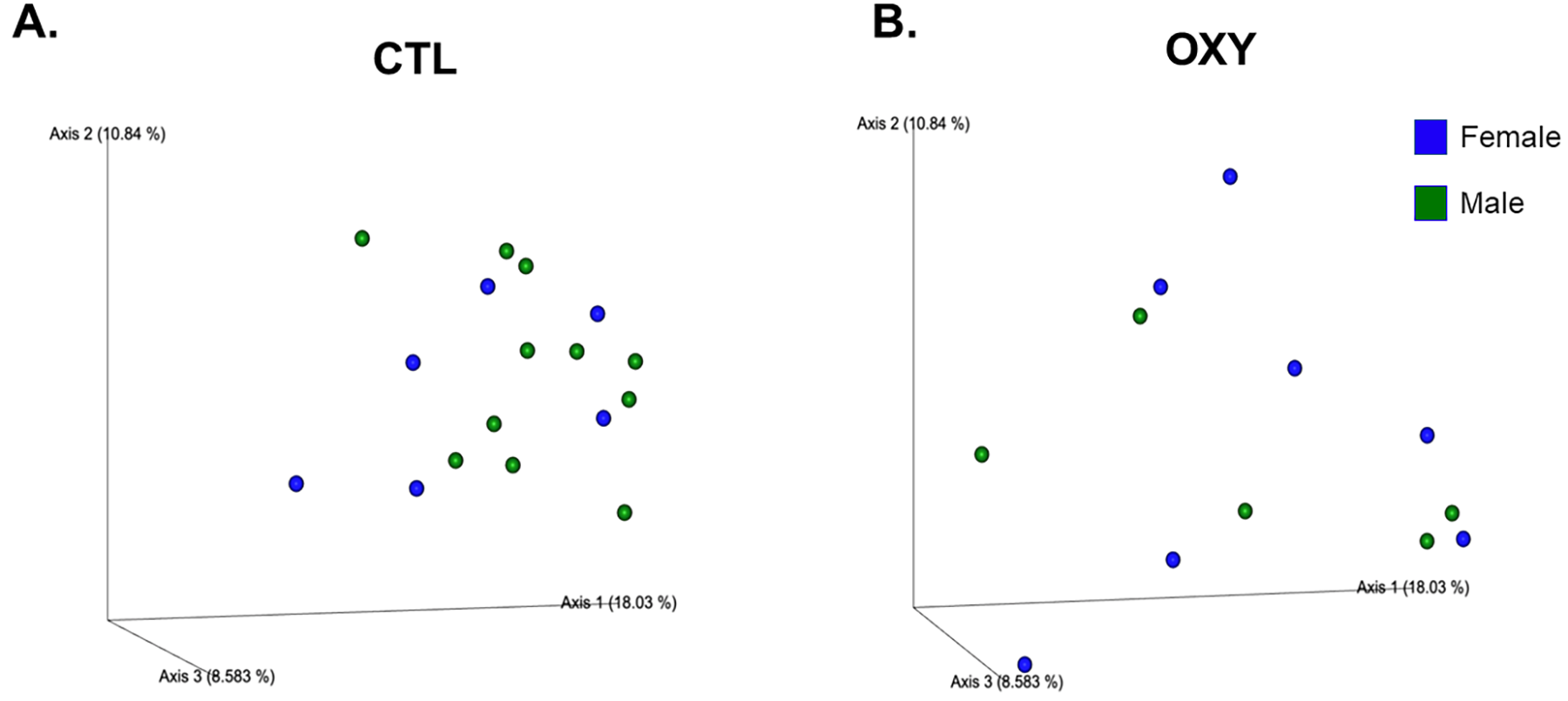

Supplement: FIG S3 [file msystems.00336-22-sf003.tif]

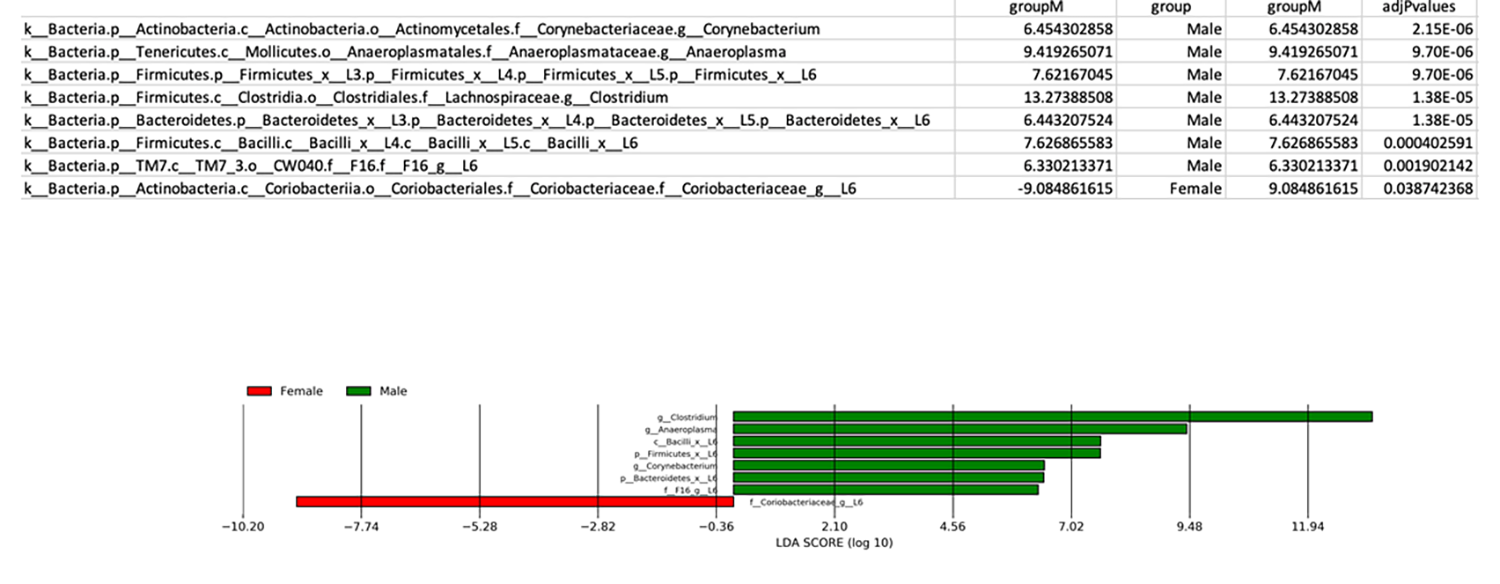

Supplement: FIG S4 [file msystems.00336-22-sf004.tif]

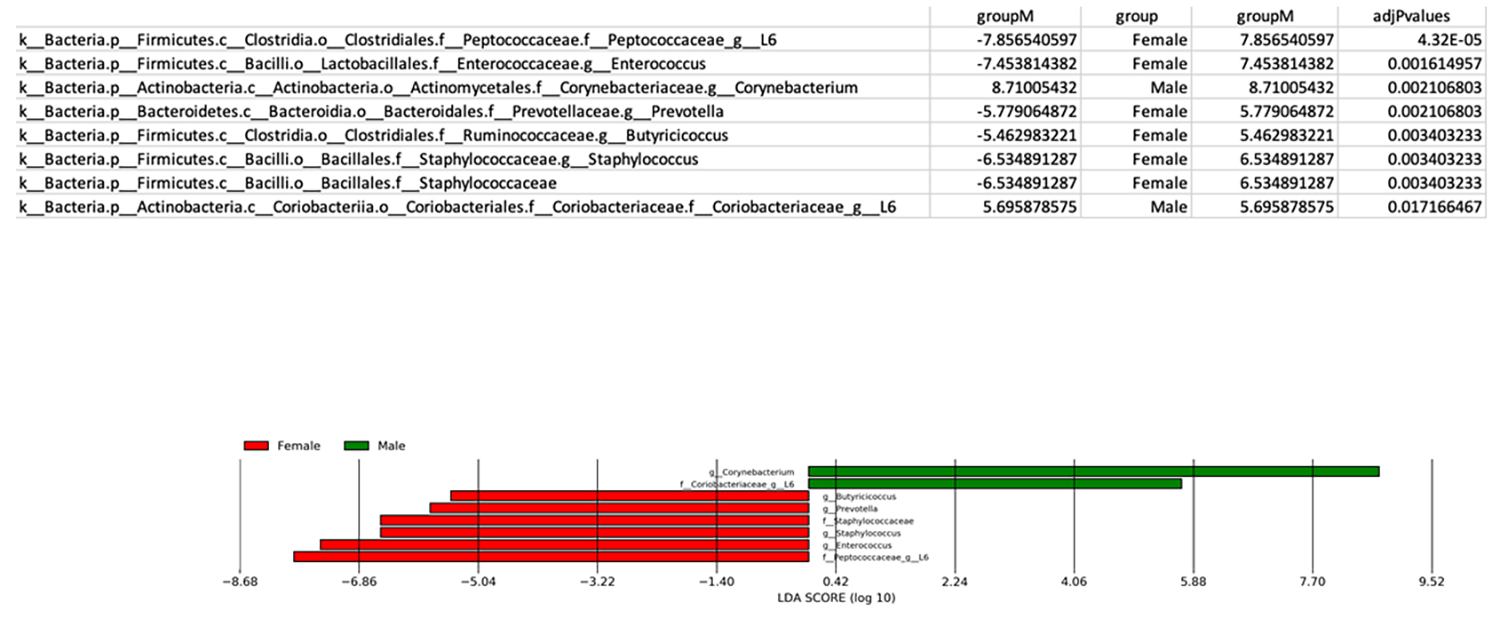

Supplement: FIG S5 [file msystems.00336-22-sf005.tif]
